# Translational Potential: Kidney Tubuloids in Precision Medicine and Regenerative Nephrology

**DOI:** 10.3390/pharmaceutics18020147

**Published:** 2026-01-23

**Authors:** Muhammad Kamal Hossain, Hwa-Young Lee, Hyung-Ryong Kim

**Affiliations:** 1 Organoids Laboratory, Department of Pharmacology, College of Dentistry, Jeonbuk National University, Jeonju 54896, Republic of Korea; kamalhossain@jbnu.ac.kr; 2 Non-Clinical Evaluation Center Biomedical Research Institute, Jeonbuk National University Hospital, Jeonju 54907, Republic of Korea; youngat84@gmail.com; 3 Department of Pharmacy, Faculty of Pharmaceutical Sciences, University of Science and Technology Chittagong (USTC), Chittagong 4202, Bangladesh; 4 Research Institute of Clinical Medicine of Jeonbuk National University—Biomedical Research Institute of Jeonbuk National University Hospital, Jeonju 54907, Republic of Korea

**Keywords:** kidney tubuloids, patient-specific phenotype, toxicity testing, personalized disease modeling, precision nephrology, regenerative medicine, translational research

## Abstract

Advances in kidney organoid technologies have expanded opportunities to model human renal development, disease, and therapeutic response. Yet pluripotent stem cell-derived organoids remain limited by cellular heterogeneity, incomplete tubular maturation and low scalability, restricting their translational relevance. Tubular-specific organoids, derived from adult kidney epithelium, address many of these constraints by providing stable, reproducible cultures enriched for functional proximal and distal tubular cells. Their polarized transport, metabolic activity and patient-specific phenotypes enable high-fidelity modeling of acute and chronic tubular disorders, nephrotoxicity, and inherited tubulopathies—areas where conventional animal and cell-line models often fall short. In this Perspective, we outline recent advances that position tubuloids as a versatile platform for drug screening, toxicity testing and personalized disease modeling. We highlight emerging integration with microfluidics, biomaterials, and gene-editing strategies that promise greater physiological realism and precision therapeutics. We also discuss persistent barriers that impede broader adoption and clinical translation. We propose a roadmap for advancing tubuloid technologies toward precision nephrology and their future incorporation into diagnostic, pharmacological and regenerative pipelines.

## 1. Introduction

The past decade has witnessed remarkable advances in in vitro kidney models, driven by the need to better understand renal development, disease mechanisms, and therapeutic interventions. Kidney organoids derived from pluripotent stem cells (PSCs) or adult stem/progenitor cells recapitulated many aspects of nephrogenesis, including the formation of glomerular and tubular compartments, and have been invaluable for modeling congenital anomalies, nephrotoxicity, and early-stage kidney disease [1]. However, conventional organoids often present limitations, including heterogeneity in cell composition, limited scalability, and incomplete maturation of tubular structures, which constrain their translational applicability.

To address these challenges, tubular-specific organoids, commonly known as tubuloids, have emerged as a refined model system. Derived from adult kidney epithelial cells, tubuloids primarily consist of proximal and distal tubular epithelium, maintaining functional characteristics such as polarized transport, active metabolic processes, and regenerative capacity [2,3]. Their reproducibility, long-term expandability, and capacity to reflect patient-specific phenotypes make them particularly attractive for high-throughput drug screening, personalized medicine, and modeling of tubular pathologies.

The clinical urgency for such advanced models is underscored by the persistent unmet needs in nephrology. For example, acute kidney injury (AKI), chronic kidney disease (CKD), and inherited or acquired tubulopathies remain major contributors to morbidity and mortality worldwide [4], with limited effective therapies beyond supportive care or transplantation. Traditional in vivo and in vitro models often fail to fully recapitulate human tubular physiology and disease progression [5], highlighting the need for translationally relevant platforms. Kidney tubuloids leverage their ability to bridge fundamental biology and clinical application. By mimicking patient-specific tubular architecture and function, tubuloids provide a promising avenue for personalized disease modeling, therapeutic screening, and regenerative strategies, offering a pathway toward precision nephrology that addresses both mechanistic insights and unmet clinical challenges.

In this Perspective, we examine the rapid evolution of kidney organoid technologies and emphasize the rising importance of tubuloids as clinically relevant tubular-specific platforms. We summarize their cutting-edge applications in disease modeling, nephrotoxicity testing, drug discovery, and regenerative strategies, while also highlighting emerging opportunities through bioengineering integration, microfluidic systems, and gene-editing-based precision therapeutics. At the same time, we address key barriers to translation, including challenges in maturation, vascular and immune incorporation, reproducibility, and regulatory standardization. By outlining both the opportunities and limitations, this article provides a clear roadmap for advancing tubuloid technologies toward precision nephrology and future clinical use. To ground this perspective in current evidence, we conducted a structured literature search covering the past 15 years (2010–2025) using PubMed, Web of Science, Google Scholar and Scopus. Search terms included kidney tubuloids, renal organoids, proximal tubule models, precision nephrology, nephrotoxicity, drug-induced kidney injury, and regenerative nephrology. The initial search results were screened by the authors through independent evaluation of titles and abstracts for relevance to human kidney biology and tubuloid-based disease modeling. We included peer-reviewed primary studies and authoritative reviews with clear experimental or translational relevance to human kidney biology. Final article selection was reached by consensus among the authors and emphasized methodological rigor, reproducibility, physiological relevance, and translational potential, with key methodological and technological advances highlighted to capture emerging trends.

## 2. Biological and Technological Foundations of Tubuloid Systems

Tubuloid technology is built on diverse yet complementary cellular sources that together provide a flexible platform for modeling renal physiology and disease. Tubuloid protocols relied on adult kidney epithelial cells isolated from nephrectomy or biopsy tissue, which retain mature tubular identity, segment-specific transporter expression, and genomic stability during long-term culture. These adult-derived tubuloids reproduce key features of proximal and distal tubular function, making them particularly valuable for nephrotoxicity assessments and solute transport studies [3,6]. In parallel, urine-derived progenitor cells, which can be collected through completely noninvasive sampling, have emerged as an important patient-specific source. These progenitors exhibit robust clonogenic potential and can be expanded into tubuloids that preserve donor-specific genetic variants and disease phenotypes [6]—thus enabling precision nephrology applications and accessible biobanking. More recently, iPSC-derived tubuloids have been developed by isolating epithelial compartments from pluripotent stem cell-derived kidney organoids and expanding them under tubuloid-promoting conditions [7]. This hybrid strategy combines the developmental flexibility and genetic tractability of iPSCs with the epithelial purity and adult-like functional characteristics of tubuloids. Together, these cellular origins support the major advantages of tubuloids over traditional primary cultures or whole iPSC-derived kidney organoids. To provide a conceptual overview of current kidney epithelial model systems, Table 1 summarizes their approximate maturation status, functional characteristics, and practical considerations. The values and ranges presented are intended as qualitative, framework-level estimates synthesizing trends reported across multiple studies and current expert consensus, rather than precise experimental benchmarks for any specific protocol.

Tubuloid cultures represent a robust platform derived from adult kidney tissue, urine epithelial cells, or patient-specific iPSCs, supporting long-term expansion while maintaining structural and genomic stability. Their patient specificity—particularly when derived from urine or iPSCs—allows faithful reproduction of individual genotypes, including rare variants and inherited tubulopathies. Importantly, tubuloids exhibit robust and physiologically relevant transporter expression, enabling accurate modeling of vectorial transport, drug secretion, and nephrotoxin sensitivity [8,9]. Furthermore, tubuloids offer significantly higher epithelial purity than whole kidney organoids, which often contain fetal mesenchymal, stromal, and off-target neuronal populations [10]. This epithelial enrichment improves reproducibility and facilitates functional assays requiring clear apical–basolateral polarity.

Rapid technological advances have further enhanced the physiological relevance of tubuloid systems. Integration with microfluidic platforms provides precise control of flow and shear stress, promoting tubular polarization, tight junction integrity, and enhanced transporter activity [11,12,13]. These organ-on-chip systems enable dynamic perfusion and real-time measurement of barrier and transport properties, thus better approximating the in vivo microenvironment. Complementary innovations include semipermeable membrane-based culture formats, such as Trans well or ECM-sandwich systems [14,15,16], which allow selective access to apical and basolateral compartments for quantitative transepithelial transport assays. Additionally, the use of extracellular vesicles (EVs) derived from mature renal cells has emerged as an efficient strategy to promote tubuloid maturation, improving metabolic competence and transport activity through EV-mediated transfer of bioactive proteins and RNAs [2,17]. Finally, gene editing technologies, particularly CRISPR/Cas9, can be applied either in donor iPSCs or directly in tubuloids, to generate isogenic disease models, functionally test pathogenic variants, and incorporation of reporter constructs for mechanistic studies [18,19]. Together, these biological foundations and engineering innovations firmly establish tubuloids as a next-generation, physiologically relevant platform for precision nephrology, drug toxicity testing, and mechanistic research into tubular function and dysfunction.

Despite these advances, several constraints remain. Inter-laboratory variability in tubuloid derivation protocols, ECM composition, and culture media contributes to heterogeneity in differentiation status and transporter expression. Donor-to-donor variation further affects baseline phenotypes and drug response profiles, complicating cross-study comparisons. Moreover, although tubuloids show structural stability, they still demonstrate incomplete functional and metabolic maturation, particularly with respect to long-term solute handling and endocrine responsiveness. Continued refinement of culture conditions, maturation cues, and standardization frameworks will be essential to overcome these limitations.

## 3. Recent Engineering Innovations for Constructing Tubuloids

**Figure 1:**
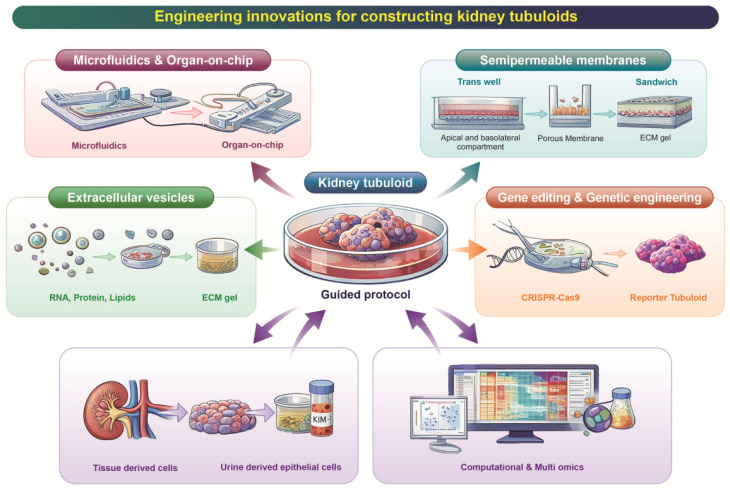
**Engineering innovations for constructing tubuloids.** This schematic summarizes recent technological strategies used to generate and refine kidney tubuloids from multiple origins, including tissue-derived cells, urine-derived epithelial cells, and induced pluripotent stem cells (iPSCs), all converging through standardized guided protocols. Key engineering innovations that enhance tubuloid formation, maturation, and functional relevance are illustrated: Microfluidics and organ-on-chip integration, enabling controlled fluid flow and physiologic microenvironmental cues; Semipermeable membrane or “sandwich” culture formats, supporting polarized epithelial organization; Extracellular vesicle-mediated maturation, promoting enhanced cellular differentiation and functional development; Gene editing and genetic engineering, allowing the creation of disease-specific or reporter tubuloids; and Computational and multi-omics approaches, facilitating deep characterization, quality benchmarking, and data-driven optimization.

Recent engineering innovations have significantly advanced the construction and functional refinement of kidney tubuloids, enabling more physiologically relevant and clinically applicable models. These emerging strategies—ranging from microfluidic integration with genetic engineering to integrative approaches—are reshaping how tubuloids are generated, matured, and scaled. Figure 1 provides an overview of these technological developments, highlighting the key approaches currently driving progress in tubuloid engineering.

### 3.1. Microfluidics and Organ-on-Chip Integration

The convergence of tubuloid technology with microfluidic and organ-on-chip platforms represents a pivotal step toward engineering physiologically faithful kidney models. Traditional static culture fails to recapitulate the biomechanical cues—particularly luminal flow and shear stress—that are essential for nephron function. In contrast, microfluidic integration enables precise modulation of flow dynamics, generating shear forces comparable to those experienced by renal tubular epithelial cells in vivo. These mechanical cues are not merely supportive; they act as powerful morphogenetic signals that drive epithelial polarization, enhance tight junction integrity, and promote correct apical–basolateral distribution of transporters such as SGLT2, OCT2, P-gp, and MRP2 [20,21,22]. Evidence from kidney-on-chip studies consistently demonstrates that applying physiological shear stress (~0.2–1 dyn/cm^2^) significantly improves barrier function, increases cilia formation, and enhances solute transport capacity relative to static conditions [23].

Moreover, microfluidic perfusion systems offer unprecedented control over the microenvironment. Tubuloid-based chips can be continuously supplied with defined media, patient-derived filtrates, or pharmacological compounds, enabling dynamic modeling of nutrient exchange, drug absorption, and tubular secretion. Integrated sensors—including TEER electrodes permit real-time monitoring of epithelial integrity and functional responses [24,25]. This continuous readout capability is particularly valuable for modeling acute kidney injury (AKI), drug-induced nephrotoxicity, and chronic disease progression, where rapid changes in barrier function or secreted biomarkers such as KIM-1, NGAL, and inflammatory cytokines serve as early indicators of cellular stress [26]. Recent demonstrations of multi-chip microphysiological systems (MPSs) incorporating tubuloids further highlight the translational advantages of this technology. These modular platforms allow compartmentalization of different nephron segments—proximal tubule, distal tubule, and collecting duct—or even integration with vascular endothelial channels to model tubulo-vascular interactions. Studies comparing MPS-integrated tubuloids to static 3D culture consistently report enhanced drug transporter function, more physiological metabolic profiles, and improved sensitivity to known nephrotoxins such as cisplatin, gentamicin, and tenofovir [27]. A notable example is the perfused iPSC-derived proximal tubule model described by Lechtenberg et al., which integrates human induced pluripotent stem cell (iPSC)-derived proximal tubule epithelial cells within a dynamic perfusion platform to emulate key physiological features of the renal tubular microenvironment. In contrast to conventional static cultures, this perfused system sustains epithelial function over extended periods and demonstrates enhanced responsiveness to clinically relevant nephrotoxins such as polymyxin B, cyclosporin A, and cisplatin. Perfusion critically shapes toxicity outcomes and biomarker profiles, including metallothionein upregulation consistent with in vivo injury. This platform also enables evaluation of nephroprotective interventions, exemplified by reduced polymyxin-induced injury with curcumin co-treatment [28]. Notably, microfluidic perfusion restores more accurate dose–response relationships, mirroring human renal handling of xenobiotics [29]. In this context, microfluidic perfusion systems and biomaterial scaffolds expand physiological realism by enabling controlled flow, polarized architecture, and sustained hormonal stimulation—highlighting the role of engineering advances as functional amplifiers, not isolated achievements.

### 3.2. Semipermeable Membrane/“Sandwich” Culture Formats

Semipermeable membrane-based culture formats—ranging from classic Transwell inserts to advanced microfabricated porous membranes—are becoming central to revealing the functional potential of tubuloids. These platforms recreate the intrinsic polarity of renal tubular epithelium by physically separating the apical and basolateral compartments. This spatial organization is not merely architectural; it directly influences vectorial transport, ion gradients, and the directionality of solute movement, all of which are hallmarks of nephron physiology. By providing selective access to both epithelial surfaces, semipermeable supports enable precise measurement of TEER, tight junction integrity, and solute flux, transforming organoid cultures into quantitatively assessable epithelial barriers [24,25].

The emergence of “sandwich” culture configurations, in which tubuloid-derived epithelial layers are positioned between extracellular matrix (ECM) gels or surrounded by a biphasic ECM–membrane interface, further enhances physiological relevance. Evidence from kidney epithelial models demonstrates that porous membranes promote enhanced polarization, more robust tight junction formation, for example, ZO-1, claudin, and occludin enrichment [30], and appropriate localization of transporters such as Na^+^/K^+^-ATPase, AQP1, OAT1/3, and SGLT2 [22]. When tubuloids are differentiated on these semipermeable substrates, they spread into 2.5D monolayers that maintain key aspects of tubular identity while achieving higher levels of functional maturity compared with suspension or dome cultures. These monolayer formats also permit controlled exposure to luminal solutes—such as glucose, albumin, or xenobiotics—while maintaining distinct basolateral composition, enabling physiologically relevant modeling of reabsorption and secretion [31].

Using sandwich formats with kidney-derived cells show improved vectorial transport of organic cations/anions, enhanced drug secretion, and the establishment of stable concentration gradients across the epithelium that mirror in vivo tubular reabsorption processes. Furthermore, sandwich cultures and semipermeable supports enable high-fidelity transepithelial transport assays. This capability is crucial for evaluating transporter-specific drug interactions, assessing nephrotoxic liability, and characterizing disease-specific changes in barrier permeability or solute transport. When combined with microfluidic perfusion, membrane-based formats support dynamic reabsorption–secretion modeling, where gradients of electrolytes, metabolites, or toxins can be established and monitored in real time. Importantly, these technologies advance tubuloids from spherical, lumen-confined organoids toward planar, accessible epithelial tissues that are compatible with standard pharmacological and toxicological assays. This transformation is key for harmonizing tubuloid platforms with regulatory testing frameworks and for facilitating comparability across laboratories.

### 3.3. Extracellular Vesicle-Mediated Maturation

Extracellular vesicles (EVs) to guide tubuloid maturation represents an emerging and highly promising strategy rooted in the kidney’s own paracrine biology. In native renal tissue, tubular epithelial cells continuously exchange molecular information through EVs—exosomes and microvesicles—that shuttle proteins, lipids, mRNAs, and regulatory microRNAs across nephron segments [32]. This vesicle-mediated communication plays a crucial role in maintaining epithelial differentiation, coordinating stress responses, and preserving transport function. Leveraging this intrinsic signaling architecture for tubuloid maturation offers a biologically grounded approach that avoids exogenous genetic manipulation. Increasing evidence supports that conditioned media or purified EVs derived from differentiated proximal tubular epithelial cells (PTECs), collecting duct cells, or other mature nephron populations can drive robust maturation programs in stem cell-derived tubuloids. Studies show that EV-treated tubuloids exhibit up-regulation of key transporters and channels, including SGLT2, OAT1/3, OCT2, AQP1, and Na^+^/K^+^-ATPase, accompanied by enhanced epithelial polarity and improved tight junction formation [2,33,34]. These molecular enhancements translate into meaningful functional improvements, such as increased albumin reabsorption, more physiologic transepithelial transport rates, and heightened sensitivity to nephrotoxins in patterns that mirror adult renal physiology. The mechanisms underpinning these changes appear to stem from the EV cargo itself.

Proteomic and transcriptomic analyses of renal EVs also reveal enrichment in differentiation-associated molecules, including HGF, BMP7, E-cadherin fragments, and microRNAs such as miR-200 family members, which are known to regulate epithelial identity and suppress mesenchymal transition [35,36,37]. When internalized by immature tubuloids, these EVs can activate pathways involved in nephron maturation, metabolic reprogramming, and cytoskeletal organization. Importantly, this mode of maturation preserves genomic integrity—an advantage compared with viral or CRISPR-based engineering—and offers a scalable, cell-free approach suitable for translational applications. Notably, experimental findings from renal regeneration and injury models reinforce the therapeutic potential of EVs [38,39,40], further supporting their capacity to induce functional differentiation. When these insights are applied to tubuloids, they underscore EVs as powerful bioactive mediators capable of bridging the developmental gap between in vitro organoids and adult kidney epithelium. As the field progresses, establishing standardized sources of renal EVs—potentially from immortalized renal cell lines, engineered producer cells, or bioreactor-expanded donor cells—will be critical. High-content profiling of EV cargo and controlled delivery systems may allow fine-tuning of maturation programs, enabling tailored tubuloids for disease modeling, drug transport studies, and personalized nephrotoxicity testing.

### 3.4. Gene Editing and Genetic Engineering

The integration of CRISPR/Cas9 gene-editing technologies into tubuloid research represents a transformative opportunity to dissect renal biology with unprecedented precision. Because tubuloids can be generated either directly from adult kidney tissue or from iPSCs, gene-editing strategies can be implemented at multiple stages of organoid development. Editing upstream in iPSCs allows the creation of isogenic pairs, in which a pathogenic variant is either introduced or corrected while retaining an identical genomic background [41]. These paired models serve as powerful tools for mechanistic studies, eliminating patient-to-patient genetic variability and thus strengthening causal inferences about genotype–phenotype relationships.

CRISPR/Cas9 can also be applied directly to established tubuloids, enabling somatic editing for target validation, pathway dissection, and therapeutic testing. This approach is particularly compelling for renal research where many disorders—such as cystic kidney diseases, tubular transport defects, or congenital tubulopathies—arise from mutations in single genes. For example, editing of PKD1/PKD2 in kidney organoids has recapitulated cystogenesis, providing a tractable system for studying polycystic kidney disease and screening anti-cystic compounds [42]. Similarly, CRISPR-mediated disruption of *SLC12A1, SLC12A3*, or *CLCNKB* in tubuloid or nephron organoid systems has been used to model Bartter and Gitelman syndromes [43,44], capturing hallmark features such as defective ion transport and altered electrolyte handling. These models offer an avenue for functional annotation of variants of uncertain significance (VUS) [45], a growing challenge in nephrogenetics.

Beyond disease modeling, gene editing enables rescue experiments, in which correction of a pathogenic mutation restores normal tubular structure or transporter function. Such studies serve as high-confidence validation that a given variant is causative, while simultaneously providing proof-of-concept evidence for emerging gene therapies. For instance, CRISPRa can upregulate endogenous protective pathways such as NRF2 or Klotho, enabling mechanistic investigation into regenerative and cytoprotective responses in kidney cells [46].

Another major advance is the creation of fluorescent and luminescent reporter lines, where transporters, injury markers, or transcriptional programs can be monitored non-invasively in real time. Reporter tubuloids expressing tagged versions of OCT2, AQP1, or stress markers such as HSP70 facilitate dynamic assessment of drug uptake, efflux kinetics, or toxic injury [47,48]. These engineered systems are particularly valuable for high-content drug screening and for studying early mechanotransductive or metabolic responses under microfluidic perfusion. Recent nephrology-focused reviews have highlighted both the successes and emerging considerations of applying CRISPR to kidney organoid and tubuloid systems. Key themes include off-target risks, mosaicism, and variability in editing efficiency depending on tubuloid maturity and cellular composition. Although these challenges are not minor, advances in high-fidelity Cas9 variants, for example, Sniper2L [49], single-cell sequencing, and long-read genotyping are rapidly improving the precision and traceability of edits in 3D kidney cultures.

### 3.5. Integrative Approaches: Computational & Multi-Omics

The intersection of tubuloids with computational biology, multi-omics, and AI offers transformative possibilities. The convergence of tubuloid technology with computational biology, multi-omics, and emerging AI frameworks is poised to redefine kidney research and translational nephrology. Comprehensive multi-omics profiling—spanning single-cell transcriptomics, proteomics, metabolomics, and epigenomics—can elucidate tubuloid identity, segment-specific programs, maturation dynamics, and disease-associated signatures, with differential expression across adult-, fetal-, and iPSC-derived models offering insights into aging processes, inflammatory pathways, and segmental specialization that can guide standardized culture optimization [50,51,52]. In parallel, integrating these datasets into in silico nephron models enables the development of renal “digital twins” that incorporate transporter kinetics, gene-expression landscapes, and metabolic flux to simulate physiological and pathological transport behaviors [53,54,55], thereby accelerating drug screening and informing the design of synthetic renal tissues. Recent advances in single-cell transcriptomics have provided powerful tools to benchmark and refine kidney organoid differentiation strategies. Wu et al. performed a comprehensive scRNA-seq analysis of human PSC-derived kidney organoids and demonstrated that these cultures contain diverse renal lineages, including podocytes and segmented tubular epithelia, alongside stromal and endothelial-like populations [56]. Importantly, their work revealed pronounced heterogeneity and the presence of off-target cell types, together with a predominantly fetal-like transcriptional profile, underscoring the developmental immaturity of current organoid systems. Comparative analysis across protocols further showed that differentiation conditions strongly influence cellular composition. By linking aberrant lineages to specific signaling pathways and applying these insights to modify culture conditions, the authors were able to reduce off-target populations and enhance nephron patterning. This study exemplifies how single-cell transcriptomics can function as a feedback framework to increase cellular fidelity, standardize organoid production, and ultimately accelerate their application in precision nephrology and regenerative medicine.

Emerging AI foundation models trained on large kidney single-cell repositories—such as Nephrobase Cell+ [57]—further extend this integrative frontier by embedding cellular states, harmonizing batch variability, and predicting lineage behavior; when coupled with tubuloid-derived data, such systems could forecast maturation trajectories, transporter induction, and injury responses entirely in silico, streamlining discovery and reducing experimental burden while charting a path toward truly predictive kidney biology.

## 4. Applications in Precision Medicine

**Figure 2:**
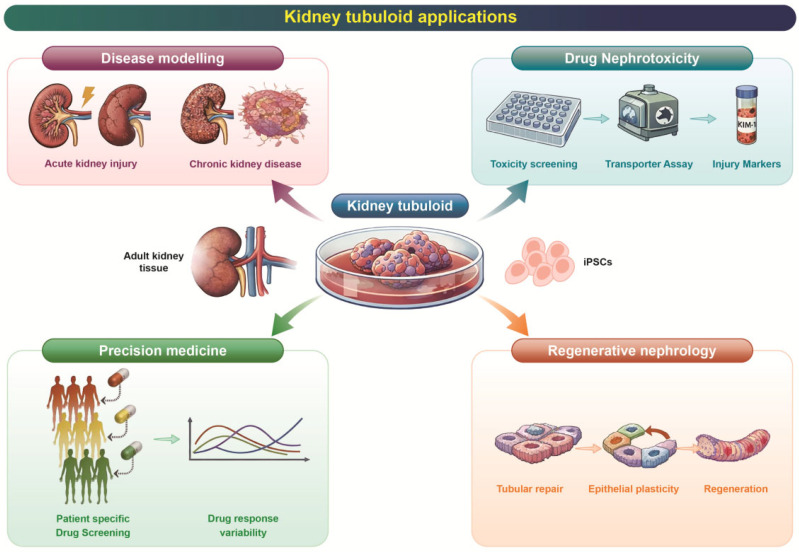
**Applications of kidney tubuloids in biomedical research and medicine.** Kidney tubuloids generated from adult kidney tissue or induced pluripotent stem cells (iPSCs) provide a versatile platform spanning multiple domains. In disease modeling, tubuloids enable investigation of acute kidney injury and chronic kidney disease mechanisms. In drug nephrotoxicity, tubuloids support toxicity screening, transporter assays, and assessment of injury biomarkers. Within precision medicine, patient-specific tubuloids allow individualized drug screening and evaluation of inter-individual variability in drug responses. In regenerative nephrology, tubuloids offer opportunities to study tubular repair, epithelial plasticity, and regenerative processes. These applications highlight the translational potential of kidney tubuloids for diagnostics, therapeutic testing, and future regenerative strategies.

The maturation of tubuloid technology, together with advances in microfluidics, biomaterials, and bioengineering, is now moving kidney research from descriptive modeling toward actionable precision medicine. In this section, engineering innovations are framed not as endpoints themselves but as enabling platforms that expand the diagnostic, prognostic, and therapeutic utility of tubuloids across patient-derived disease modeling, regenerative nephrology, and bioartificial organ design (Figure 2). When coupled with clinical metadata, multi-omics profiling, and functional assays, tubuloids provide a bridge between mechanistic discovery and individualized patient care.

Recent advances in adult stem cell-derived kidney tubuloids have markedly expanded the scope of patient-specific renal modeling. Schutgens et al. demonstrated that tubuloids can be robustly generated from both human kidney tissue and even from urine, enabling minimally invasive access to patient material. These cultures predominantly represent the tubular epithelium, retain segment-specific marker expression, and can be expanded long-term while preserving genomic stability. Importantly, patient-derived tubuloids conserve disease-associated genotypes and phenotypes, including defective cystic fibrosis transmembrane conductance regulator (CFTR) activity in cystic fibrosis, allowing functional assays and drug-response testing in a personalized manner. Their physiologically relevant transporter activity and sensitivity to nephrotoxic compounds further support applications in drug screening and toxicity assessment [6]. These findings position tubuloids as a powerful platform for precision nephrology, living biobanking, and translational research aimed at individualized diagnosis and therapy.

### 4.1. Patient-Derived Disease Modeling

Because tubuloids can derive from adult kidney biopsies, urine, or even iPSC lines from patients, they offer a direct route to patient-specific disease modeling [10,58]. For example, CD24^+^-derived tubuloids have been used to model autosomal dominant polycystic kidney disease (ADPKD) [58]. Yaoxian Xu et al. demonstrated that CRISPR–Cas9 knockout of PKD1 or PKD2 induced cyst formation, faithfully recapitulating a hallmark feature of ADPKD, while treatment with tolvaptan reduced cyst size in a genotype-specific manner. Notably, PSC-derived organoid models did not exhibit the same pharmacologic response [58], underscoring that patient-derived tubuloids preserve clinically relevant drug sensitivities. As biobanking and bioengineering strategies increase scalability, such patient-specific tubuloid systems will increasingly support precision diagnostics and therapeutic stratification.

### 4.2. Toward Regenerative Nephrology

Tubuloids provide a experimentally tractable platform to study nephrotoxicity, injury responses, and the dynamics of tubular repair, thereby informing regenerative nephrology strategies. Recent studies have demonstrated that tubuloids derived from human renal tubular epithelial cells faithfully recapitulate key features of drug-induced kidney injury [59,60]; for example, Nakao et al. showed that cisplatin treatment induces DNA damage marked by γH2AX, upregulation of the injury biomarker KIM-1, apoptosis via cleaved caspase-3, and early epithelial–mesenchymal transition reflected by increased vimentin expression [60]. Prolonged or repeated exposure further drives a senescent phenotype characterized by p16/p21 expression, senescence-associated secretory signaling, and fibrosis-like remodeling, paralleling chronic kidney disease progression [60,61]. Morizane and co-workers provided an illustrative example of how kidney organoids can be leveraged to model clinically relevant toxic injuries. By exposing 3D hiPSC-derived kidney organoids to gentamicin and cisplatin, they demonstrated that these systems not only respond to nephrotoxic insults but do so in a segment-specific manner. Gentamicin treatment led to a clear up-regulation of KIM-1 along the luminal surface of LTL-positive proximal tubules, whereas Cadherin-1-positive distal tubules were largely unaffected. Complementary gene-expression analyses confirmed a dose-dependent rise in KIM-1, reinforcing the notion that proximal tubules are preferentially injured. Cisplatin elicited a similar injury signature, characterized by KIM-1 induction together with suppression of Cadherin-1 [62], thereby highlighting toxicity that spans both proximal and distal tubular compartments. Extending this concept, Bajaj and colleagues developed more complex, multicellular 3D kidney constructs containing both proximal tubule cells and podocytes to evaluate their usefulness as nephrotoxicity screening platforms. These models were able to discriminate between classes of nephrotoxic agents: classical tubular toxins such as gentamicin, citrinin, cisplatin, and rifampicin triggered increases in tubular injury markers KIM-1 and HO-1, while glomerular toxins including doxorubicin and puromycin preferentially elevated podocyte-associated markers such as NPHS1 and WT1 [63]. Together, these studies underscore the growing potential of hiPSC-derived kidney organoids to capture segment-specific injury responses and to serve as human-relevant testbeds for nephrotoxicity assessment in a precision medicine framework.

Beyond modeling toxicity, tubuloids also offer an experimentally accessible window into the kidney’s intrinsic but limited regenerative capacity; their retained proliferative potential, epithelial polarity, and responsiveness to extracellular vesicles or growth factors make them ideal for dissecting the molecular programs governing dedifferentiation, redifferentiation, and tissue repair. Importantly, engineering innovations act as enabling tools here: microfluidic platforms, extracellular matrix-tuned biomaterials, and exosome/EV delivery systems allow controlled induction of injury and regeneration in tubuloids. By inducing injury through agents like cisplatin, hypoxia, mechanical stress, or cytokine stimulation and subsequently applying regenerative cues such as EVs or small molecules, researchers can dissect whether tubules undergo adaptive repair or evolve toward maladaptive fibrosis. These integrated bioengineering approaches therefore accelerate both mechanistic insight and therapeutic discovery.

### 4.3. Modeling Complex Pathophysiology

Beyond monogenic disease and injury, tubuloids are increasingly capable of modeling multifaceted renal physiology and transport disorders. For instance, collecting duct-derived tubuloids have demonstrated regulated water transport: a recent study reported endogenous expression of aquaporin 2 (AQP2) and vasopressin-2 receptor (V2R), responsiveness to desmopressin, and a measurable swelling response in 3D culture [3,64,65]. A recent study by Olde Hanhof et al. describes the successful generation and differentiation of mouse kidney tubuloids into a collecting duct (CD)-enriched phenotype, providing an innovative in vitro platform to explore CD physiology. In this model, tubuloids were cultured from primary renal epithelial cells and conditioned with a specialized medium to enhance expression of key collecting duct markers such as AQP2, AQP3, and subunits of the epithelial sodium channel (ENaC). Notably, differentiated tubuloids exhibited polarized expression of these proteins and elicited physiological responses to desmopressin and forskolin, including apical translocation of AQP2, indicating functional maturation toward a CD profile. Functional assays further demonstrated amiloride-sensitive sodium transport, reflecting ENaC-mediated electrolyte handling characteristic of the native collecting duct [65]. This model overcomes several inherent limitations of traditional immortalized cell lines by maintaining more physiologically relevant expression and polarization of key transport mechanisms, thereby enabling more accurate studies of electrolyte and water regulation. Despite some heterogeneity in segment identity within the cultures, this tubuloid system represents a significant advance in kidney organoid technology and complements existing organoid and in vivo models for dissecting CD physiology and disease mechanisms. These findings suggests that tubuloids could model rare water-balance disorders for example, nephrogenic diabetes insipidus, electrolyte imbalance syndromes, and multifaceted transport pathologies.

### 4.4. Biobanks for Precision Testing

One of the most exciting translational opportunities is the creation of tubuloid biobanks [8]. By deriving tubuloids from diverse patient cohorts including CKD, genetic kidney disease, different ethnicities, could develop a repository could captures inter-individual variability in drug responses, transporter expression, and injury susceptibility. When linked to clinical metadata, multi-omics readouts, and high-throughput engineering platforms, such biobanks would enable- personalized nephrotoxicity testing, dose optimization for nephrotoxic drugs, prediction of adverse events and responder subgroups and stratification for regenerative or antifibrotic therapies. Thus, the combination of biobanking, engineering scalability, and functional readouts directly advances precision nephrology.

**Figure 3:**
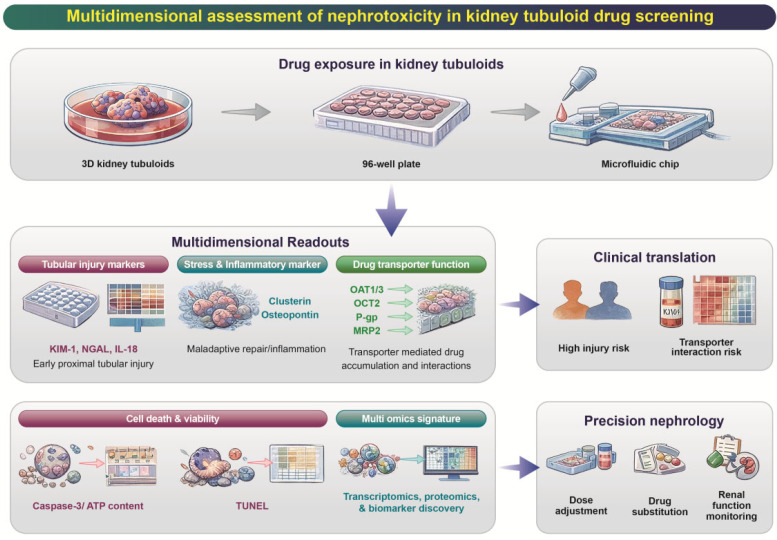
**Multidimensional assessment of nephrotoxicity using kidney tubuloid-based drug screening platforms.** Kidney tubuloids cultured in various formats—including 3D culture dishes, multiwell plates, and microfluidic chips—can be exposed to candidate drugs to evaluate nephrotoxic potential. Drug exposure enables acquisition of multidimensional readouts, including tubular injury biomarkers (e.g., KIM-1, NGAL, IL-18) indicative of early proximal tubular injury, stress and inflammatory markers such as clusterin and osteopontin reflecting maladaptive repair and inflammation, and drug transporter function (OAT1/3, OCT2, P-gp, MRP2) representing transporter-mediated drug accumulation and interactions. Additional measurements of cell death and viability (caspase-3 activity, ATP content, TUNEL staining) and multi-omics signatures (transcriptomics, proteomics, biomarker discovery) provide deeper mechanistic insight.

Moreover, renal tubuloid technology has also emerged as a powerful platform for experimental and preclinical assessment of drug-induced nephrotoxicity, representing a key application within precision medicine. Beyond the frequently reported KIM-1, tubuloid-based models enable the evaluation of a broader panel of injury, stress, and functional markers that reflect clinically relevant mechanisms of renal toxicity. These include NGAL, CLU, SPP1, and IL-18, which are associated with tubular stress, inflammation, and epithelial damage [66]. In parallel, functional readouts such as proximal tubule transporter expression and activity (e.g., OAT1/3, OCT2, P-gp), epithelial barrier integrity (tight junction proteins such as ZO-1 and occludin) cell polarity, and viability or apoptosis markers (e.g., caspase-3 activation) are commonly assessed [64,67,68]. Importantly, transcriptomic, proteomic, and secretome-based profiling of tubuloids enables mechanism-specific toxicity signatures, allowing discrimination between mitochondrial toxicity, transporter-mediated drug accumulation, and inflammatory or fibrotic responses. These multi-parametric endpoints position renal tubuloids as a physiologically relevant and scalable system for drug screening, nephrotoxicity prediction, and patient-tailored safety assessment in precision nephrology (Figure 3).

To highlight how different biomarkers contribute to individualized therapeutic strategies, Table 2 outlines the key categories, markers/readouts, indications, and their relevance for precision medicine.

### 4.5. Bioartificial Kidney Engineering

Efforts to construct bioartificial kidneys have often focused on hollow-fiber devices seeded with renal epithelial cells [69]. Incorporating tubuloids—particularly EV-matured models forming polarized monolayers with cilia and functional transporters such as OAT1 [2] offers the potential to markedly enhance physiological relevance. Embedding tubuloid-derived epithelia into engineered scaffolds and microfluidic architectures could yield units capable of regulated filtration, reabsorption, and secretion, addressing major limitations of current renal replacement strategies.

However, therapeutic implantation remains a major challenge. Tubuloids currently lack vasculature, immune components, and the supportive architecture of a native nephron. For translation, strategies are needed to vascularize tubuloid grafts, ensure immunocompatibility, and integrate with host tissue. Future progress will depend on convergent engineering approaches, including vascularization strategies, stromal co-culture systems, immunocompatible biomaterials, and dynamic perfusion devices. These innovations do not replace biological inquiry but instead enable the construction of more physiologically relevant and functionally competent renal constructs.

## 5. Challenges & Considerations for Clinical Translation

Despite the rapid evolution of tubuloid technology and its growing promise for personalized nephrology, drug development, and regenerative medicine, several critical hurdles must be addressed before these systems can be deployed widely in clinical or regulatory settings. The path to translation will require coordinated scientific, technical, regulatory, and ethical efforts, as well as a commitment to building shared infrastructures that support reproducibility and patient safety.

### 5.1. Incomplete Functional Maturation and Segmental Representation

One of the most pressing challenges is the functional maturity and physiological relevance of kidney tubuloids. Although advances such as extracellular vesicle (EV)-mediated stimulation and microenvironmental modulation have substantially improved tubuloid maturation, these models still do not fully replicate the functional, structural, and biomechanical complexity of in vivo renal tubules. Attaining uniform and physiologically relevant expression of key transporters—including both apical proteins such as SGLT2, NHE3, MATE1 and basolateral proteins such as Na^+^/K^+^-ATPase, OAT1/3, OCT2—remains a central challenge. Static cultures, in particular, fail to reproduce critical biomechanical cues such as luminal shear stress, which is known to regulate primary cilium length, tight junction formation, and flow-dependent gene expression. Additionally, tubuloids lack native gradients of oxygen, metabolites, and electrolytes that shape real nephron physiology. The absence of an in vivo-like stromal function, vasculature, and immune environment further limits tubuloid maturation and disease modeling fidelity. From a future perspective, achieving higher physiological fidelity will likely demand multimodal innovations, combining microfluidic perfusion, engineered ECM scaffolds, endothelial or mesenchymal co-culture, and biophysical stimulation such as cyclic strain.

### 5.2. Model Complexity

Although tubuloids provide an experimentally tractable disease model, donor-to-donor variability remains a notable limitation, introducing additional complexity to data interpretation. Tubuloids derived from aged, chronically diseased, or genetically predisposed kidneys may display intrinsic differences in proliferative potential, metabolic state, and responses to drugs or toxins. Preliminary data suggest that tubuloids derived from adult kidneys already manifest inflammatory signatures [10], and given what is known about mitochondrial dysfunction and epithelial injury in CKD, it is plausible that tubuloids from healthy versus CKD donors may differ substantially. This underscores the need for careful donor characterization in future tubuloid-based disease models. To overcome these obstacles, the field must converge on consensus guidelines for protocol standardization, common quality control (QC) metrics—such as validated assays for epithelial polarity, ciliary morphology, transporter localization and functional readouts—and the establishment of reference tubuloid lines or benchmark datasets. Furthermore, tubuloids may carry unique genomic information linked to patient identity, robust data protection frameworks are needed. Genetic modification: CRISPR-mediated editing of tubuloids for disease modeling or functional enhancement introduces additional ethical scrutiny, especially when modifications could theoretically affect downstream therapeutic applications.

### 5.3. Technical and Scalability Challenges

Another concerning challenge is the significant inter-laboratory variability in tubuloid generation and maintenance. Differences in derivation methods—whether from urine, renal tissue, or iPSC-derived intermediate nephron progenitors—can produce organoids with divergent growth kinetics and functional maturity. Media formulations frequently differ across labs, with variations in growth factors such as EGF, Noggin, R-spondin, small molecules, and batch-to-batch differences in basement membrane. Similar variability is observed in passage timing, splitting ratios, and differentiation protocols, all of which contribute to heterogeneity in morphology, polarity, and transporter expression.

### 5.4. Translational and Regulatory Considerations

As tubuloid technologies advance toward personalized medicine, ensuring equitable access for populations with limited healthcare resources remains a major societal concern. Priority should be given to establishing global tubuloid biobanks that capture the genetic, clinical, and environmental diversity of kidney disease—ranging from CKD and monogenic disorders to healthy controls—integrated with robust clinical metadata and genomics to create standardized reference panels. From a translational standpoint, movement toward clinical application will require clearly defined regulatory pathways. Issues such as batch-to-batch variability, long-term stability, sterility, and functional qualification of tubuloids must be addressed through standardized manufacturing and quality-control frameworks. Harmonization of protocols across laboratories will be essential to enable reproducibility, cross-study comparison, and eventual regulatory acceptance. Ethical and governance considerations also become increasingly prominent. The derivation of tubuloids from patient samples necessitates transparent consent processes, clarity regarding secondary use, and protection of privacy when clinical and genomic data are linked. Data-sharing frameworks will need to balance open science with appropriate safeguards and respect for donor autonomy.

## 6. Conclusions

Looking ahead, a coordinated roadmap is essential to unlock the full translational potential of tubuloids and position them as a cornerstone of future nephrology research and regenerative medicine. Future progress should be centered on advancing maturation platforms such as scaling extracellular vesicle-based approaches, developing microfluidic “tubuloid-on-chip” systems with physiological gradients, and integrating endothelial or stromal co-cultures. Multi-omics and AI-driven frameworks, along with in silico digital twins, can guide optimization, while rigorous preclinical testing will ensure safety, function, and injury resilience. Close regulatory engagement will be essential to establish standards and enable the use of tubuloids for precision-medicine assays and, ultimately, therapeutic renal reconstruction.

## Figures and Tables

**Table 1:** Comparative functional characteristics of kidney tubuloids, PSC-derived kidney organoids, and native human kidney tissue.

Model System	Primary Source	Transporter Expression *	Barrier Function (TEER) *	Injury/Stress Marker Response	Maturation Level	Advantages	Limitations
Native human kidney	Adult renal tissue	Reference standard (100%) for OCT2, OAT1/3, MATE1/2-K, P-gp	High, physiological (>1000 Ω·cm^2^, segment-dependent)	Robust, clinically validated (KIM-1, NGAL)	Fully mature	Standard physiology	Limited availability; non-scalable
Tissue-derived kidney tubuloids	Adult kidney biopsy	High and stable; ~50–90% of adult levels for key transporters	Moderate–high (≈200–800 Ω·cm^2^)	Strong KIM-1, NGAL induction after toxic/ischemic stress	High	Closest in vitro adult tubular phenotype	Donor variability; no vasculature/immune cells
Urine-derived kidney tubuloids	Exfoliated renal epithelial cells	Moderate–high; comparable to tissue-derived tubuloids for proximal markers	Moderate (≈150–600 Ω·cm^2^)	Reproducible KIM-1, NGAL responses	Intermediate–high	Non-invasive, repeat sampling	Heterogeneous starting material
iPSC-derived kidney tubuloids	Differentiated hiPSCs	Moderate; higher than PSC organoids but below adult tissue	Low–moderate (≈50–300 Ω·cm^2^)	Detectable injury responses; lower dynamic range	Intermediate	Scalable; genetically editable	Incomplete maturation
PSC-derived kidney organoids	hESCs/hiPSCs	Low–moderate; fetal-like transporter profiles	Low (<100 Ω·cm^2^)	Injury marker induction inconsistent	Low–intermediate	Developmental modeling	Immaturity; batch variability

* Transporter expression levels and trans-epithelial electrical resistance (TEER) ranges are presented as approximate qualitative ranges based on reported trends across the literature and collective expert interpretation. Values may vary substantially across protocols, laboratories, donors, and assay formats and should not be interpreted as definitive quantitative measurement.

**Table 2:** Nephrotoxicity markers and functional endpoints assessed in renal tubuloid-based drug screening platforms.

Category	Markers/Readouts	Indication	Relevance for Precision Medicine
Tubular injury markers	KIM-1, NGAL, IL-18	Proximal tubule injury and stress response	Early and sensitive detection of drug-induced kidney injury
Stress and inflammation markers	CLU, SPP1, cytokine release	Cellular stress, inflammation, maladaptive repair	Mechanistic stratification of nephrotoxic responses
Transporter expression & function	OAT1, OAT3, OCT2, P-gp, MRP2	Drug uptake/efflux and accumulation	Prediction of transporter-mediated nephrotoxicity and drug–drug interactions
Barrier and epithelial integrity	ZO-1, occludin, epithelial polarity	Tubular barrier disruption and loss of differentiation	Assessment of functional epithelial damage
Cell death and viability	Caspase-3 activation, TUNEL, ATP content	Apoptosis and cytotoxicity	Quantification of dose-dependent toxicity
Multi-omics injury signatures	Transcriptomics, proteomics, secretome profiling	Pathway-level toxicity mechanisms	Personalized toxicity profiling and biomarker discovery
Functional physiology	Transport assays, metabolic activity	Tubular functional competence	Improved translational relevance compared to 2D cultures

## Data Availability

The study is based solely on previously published literature. No datasets were generated or analyzed. All sources of information are cited in the reference list.

## Abbreviations

The following abbreviations are used in this manuscript:

SGLT2	Sodium-glucose cotransporter 2
OCT2	Organic cation transporter 2
P-gp	P-glycoprotein
MRP2	Multidrug resistance-associated protein 2
KIM-1	Kidney injury molecule-1
LTL	Lotus tetragonolobus lectin
HO-1	Heme oxygenase-1
NPHS1	Nephrin-1
WT1	Wilms’ tumor 1
NGAL	Neutrophil gelatinase-associated lipocalin.
CLU	Clusterin
SPP1	Secreted Phosphoprotein 1/Osteopontin
ZO-1	Zonula occludens-1.
Na^+^/K^+^-ATPase	Sodium-potassium-exchanging adenosine triphosphatase
AQP1	Aquaporin-1
OAT1/3	Organic anion transporter-1/3
HGF	Hepatocyte growth factor
BMP7	Bone morphogenetic protein 7
miR-200	microRNA-200
PKD1/2	Polycystic kidney disease 1/2
SLC12A1/3	Solute carrier family 12-member 1/3 (Na-K-Cl cotransporter)
CLCNKB	Chloride channel, kidney, B
VUS	Variants of uncertain significance
NRF2	Nuclear factor erythroid 2-related factor 2.
HSP70	Heat shock protein 70
CD24^+^	Cluster of differentiation 24 positive cell
γH2AX	Gamma H2A histone family member X.
p16	Cyclin-dependent kinase inhibitor 2A
P21	Cyclin-dependent kinase inhibitor 1A
NHE3,	Sodium-hydrogen exchanger 3
MATE1	Multidrug and toxin extrusion protein 1
